# Supplementary material for: Peering into lunar permanently shadowed regions with deep learning
Source: Nat Commun. 2021 Sep 23;12:5607. doi: 10.1038/s41467-021-25882-z (PMC8460740; doi:10.1038/s41467-021-25882-z)
Supplement: Supplementary file 1 — Supplementary Information [file 41467_2021_25882_MOESM1_ESM.pdf]

Supplementary Information

Peering into Lunar Permanently Shadowed Regions with Deep Learning

Bickel, V.T.[1]*; Moseley, B.[2]; Lopez-Francos, I.[3]; Shirley, M.[3]

[1] Max Planck Institute for Solar System Research, Göttingen, GER
[2] University of Oxford, Oxford, UK
[3]NASA Ames Research Center, Mountain View, USA

Corresponding author: Bickel, V.T. (bickel@mps.mpg.de)

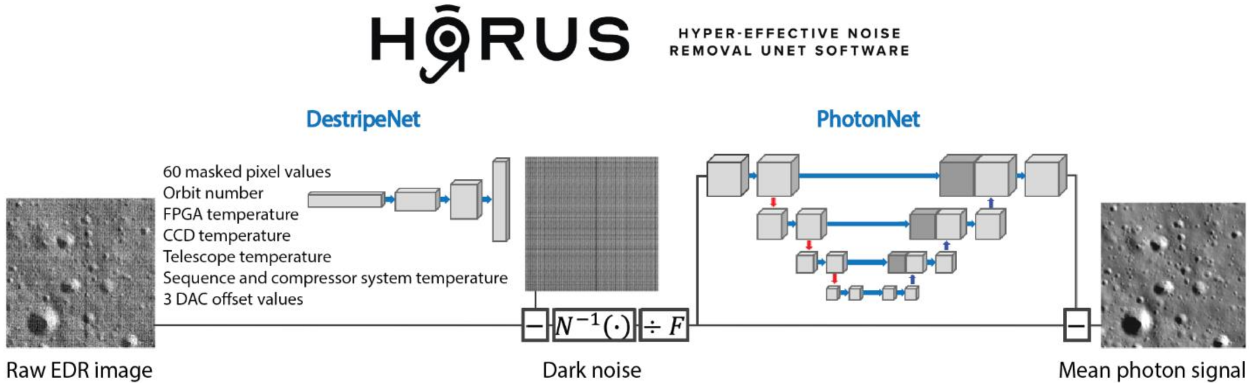

Figure S1 | HORUS workflow. HORUS uses two deep neural networks applied in sequence to remove different noise sources in raw NAC EDR images. The first network, called DestripeNet, takes a set of meta data recorded at the time of image capture as input (listed in the plot), and outputs a prediction of the CCD dark noise contained in the image. This is subtracted from the input image, and the nonlinearity and flatfield corrections (as used in the current NAC Isis3 calibration routine) are applied. The second network, called PhotonNet, estimates residual noise sources in this image (such as photon (shot) noise and CCD read noise) and outputs an estimate of the mean photon signal contained in the input image. Modified from [24]. Raw NAC image credits to NASA/LROC/GSFC/ASU.

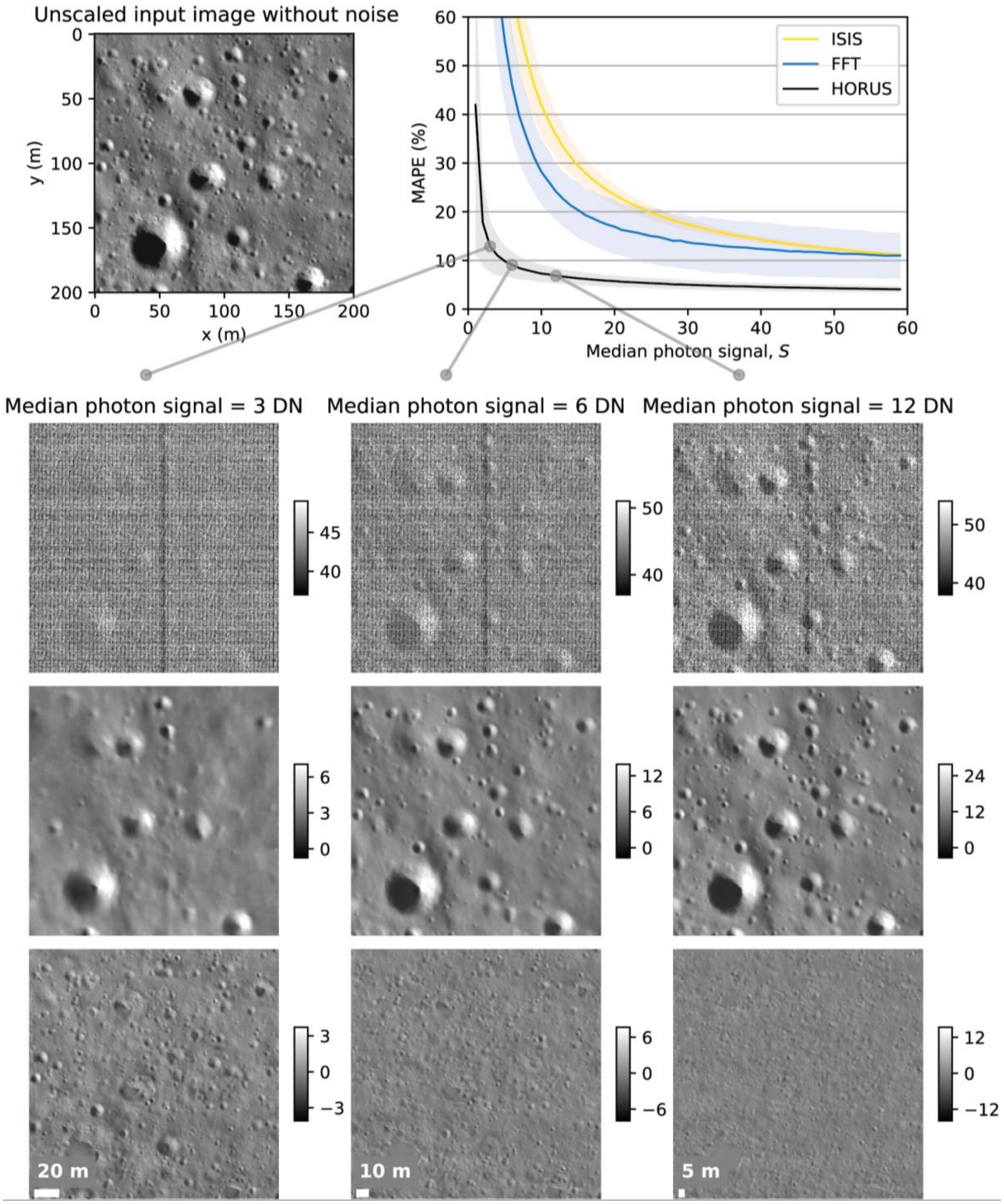

Figure S2 | Denoising performance of HORUS with varying photon signal levels. We observe, on synthetic data, that the minimum feature size resolvable by HORUS depends strongly on the photon signal level. An example sunlit NAC image (top left) is rescaled to different low median photon counts and synthetic noise is added using HORUS' physical noise model of the NAC CCD (top row of the image grid). The application of HORUS and its difference from the input image are plotted (middle and bottom rows of the image grid). For median photon signals below ~12 DN (digital number) feature sizes less than ~5 m are not recovered by HORUS. Top right shows the mean absolute percentage error (MAPE) of HORUS over a set of 150,000 synthetic test images binned by the median photon signal in the image, compared to the standard NAC calibration routine (Isis3) and a standard FFT (Fast Fourier Transform) denoising algorithm. Figure modified from [24]. Raw NAC image credits to NASA/LROC/GSFC/ASU.

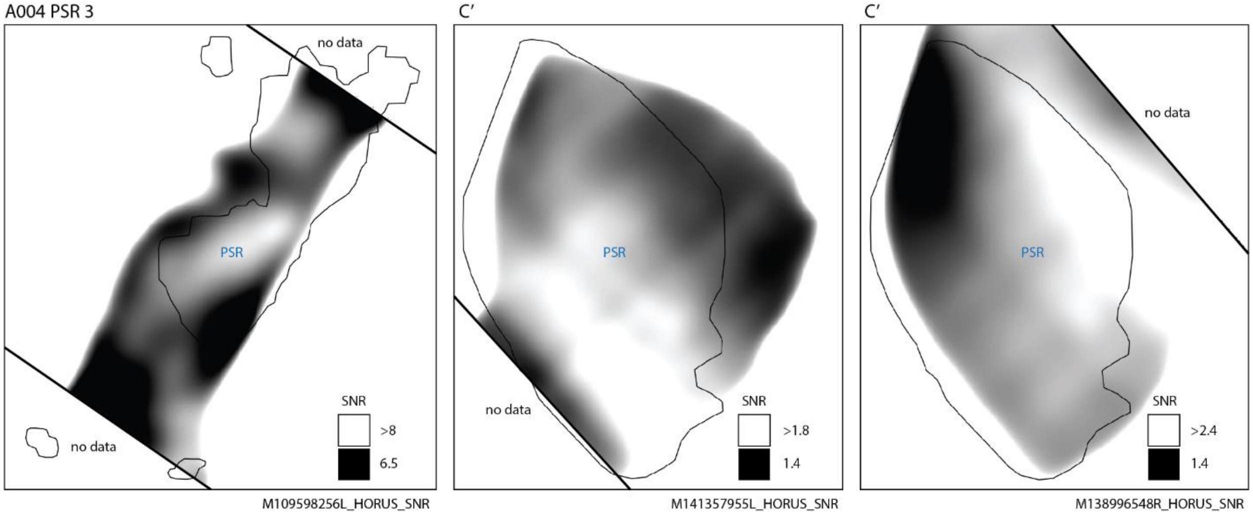

Figure S3 | SNR maps of A004 and C′. Example SNR (signal to noise ratio) maps for three HORUS images covering AoI PSRs A004 PSR 3 and C′ (outlined in black). Color scales are optimized for the SNR values within the PSRs, i.e., outside of PSRs values are only given as > max. intra-PSR value. These maps show how SNR varies laterally and between AoIs and images. Raw NAC image credits to NASA/LROC/GSFC/ASU.

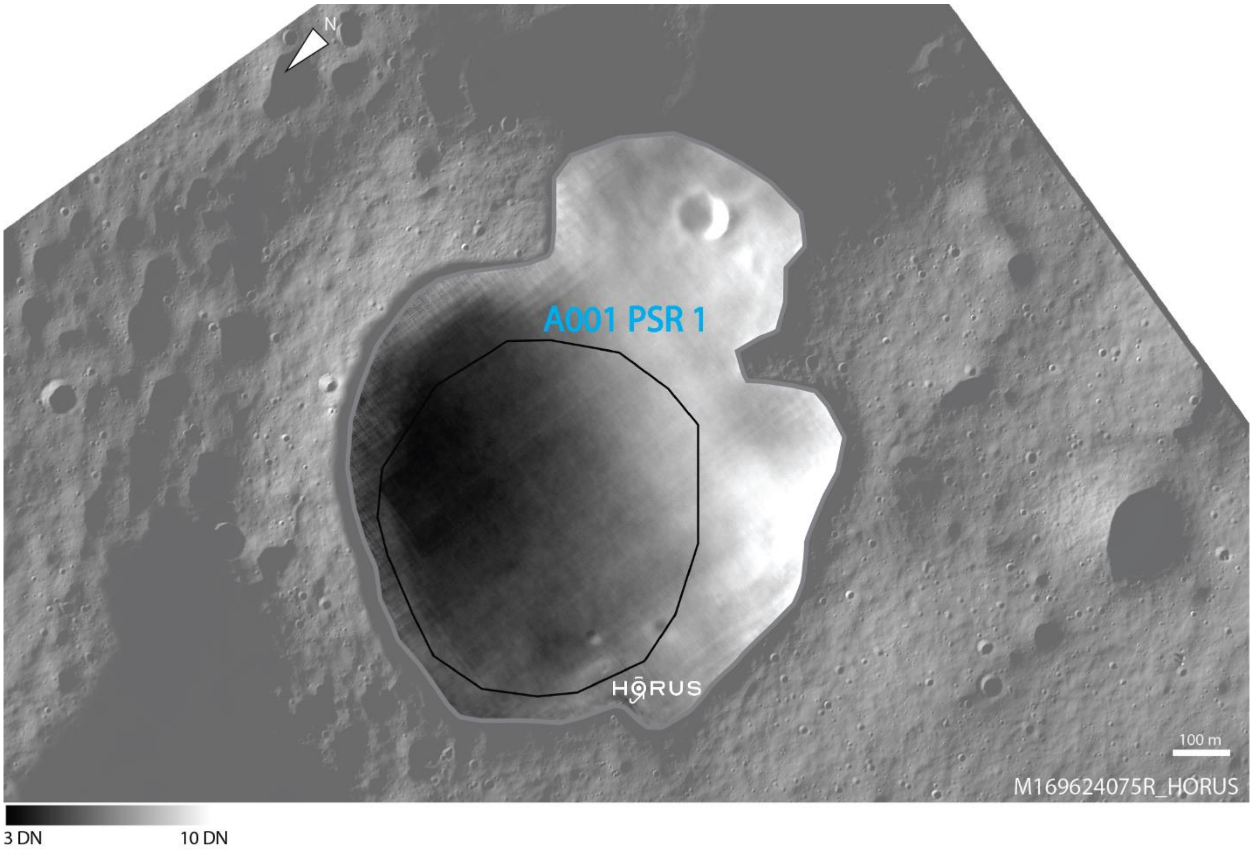

Figure S4 | AoI 001 PSR 1. View of the PSR (indicated by the black outline), NAC-observed TSR (indicated by the gray outline), and the sunlit surroundings. The most appropriate, currently available HORUS frame has been used to produce this figure. The sunlit part of the image is slightly transparent to help showcase the shadowed region. Raw NAC image credits to NASA/LROC/GSFC/ASU.

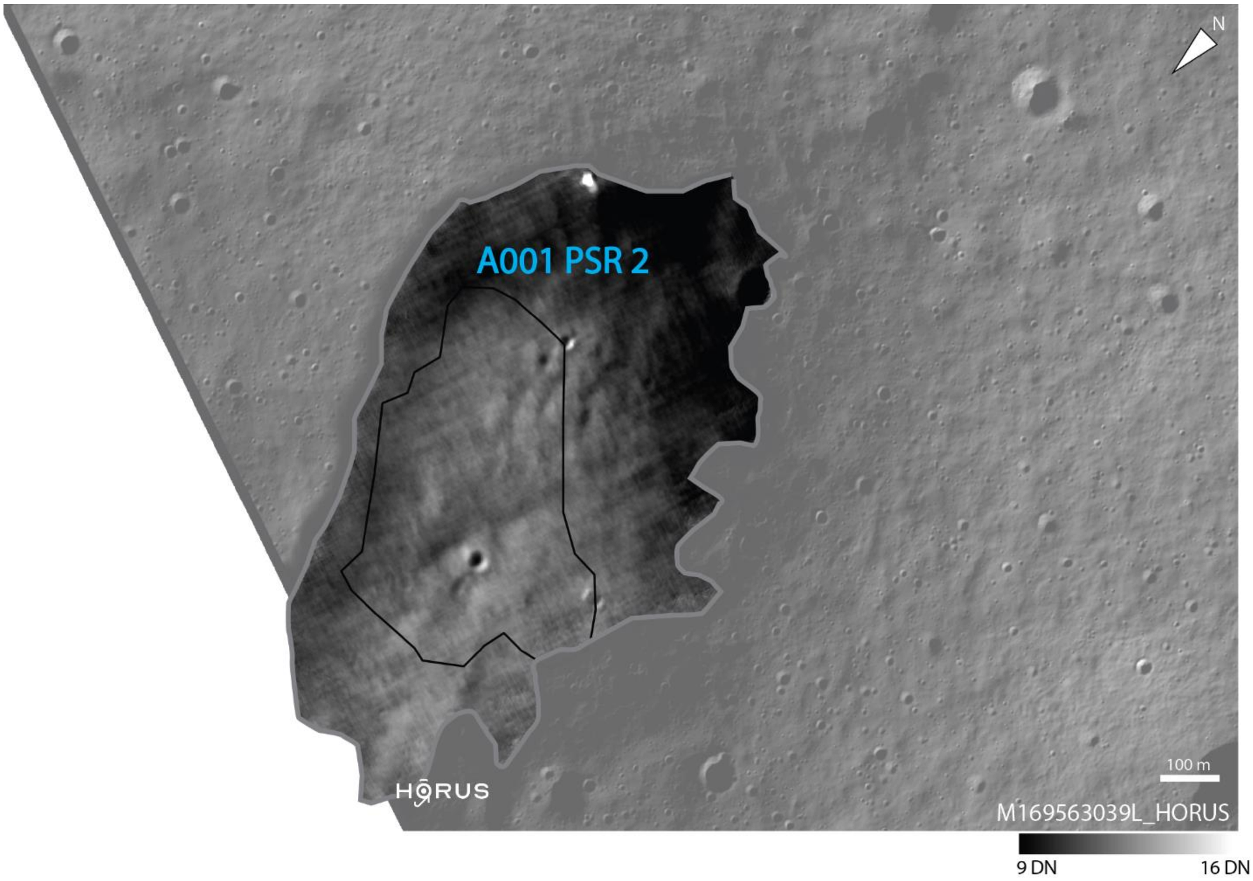

Figure S5 | AoI 001 PSR 2. View of the PSR (indicated by the black outline), NAC-observed TSR (indicated by the gray outline), and the sunlit surroundings. The most appropriate, currently available HORUS frame has been used to produce this figure. The sunlit part of the image is slightly transparent to help showcase the shadowed region. Raw NAC image credits to NASA/LROC/GSFC/ASU.

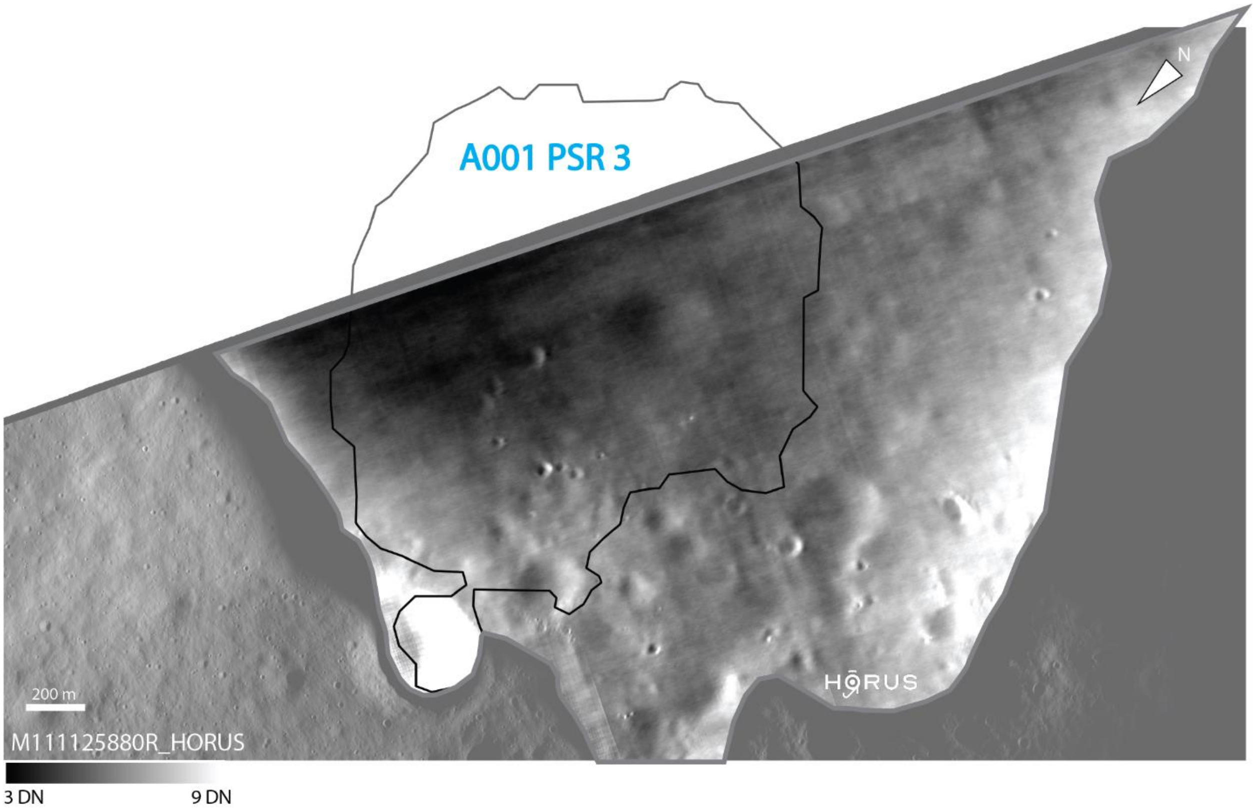

Figure S6 | AoI 001 PSR 3. View of the PSR (indicated by the black outline), NAC-observed TSR (indicated by the gray outline), and the sunlit surroundings. The most appropriate, currently available HORUS frame has been used to produce this figure. The sunlit part of the image is slightly transparent to help showcase the shadowed region. Raw NAC image credits to NASA/LROC/GSFC/ASU.

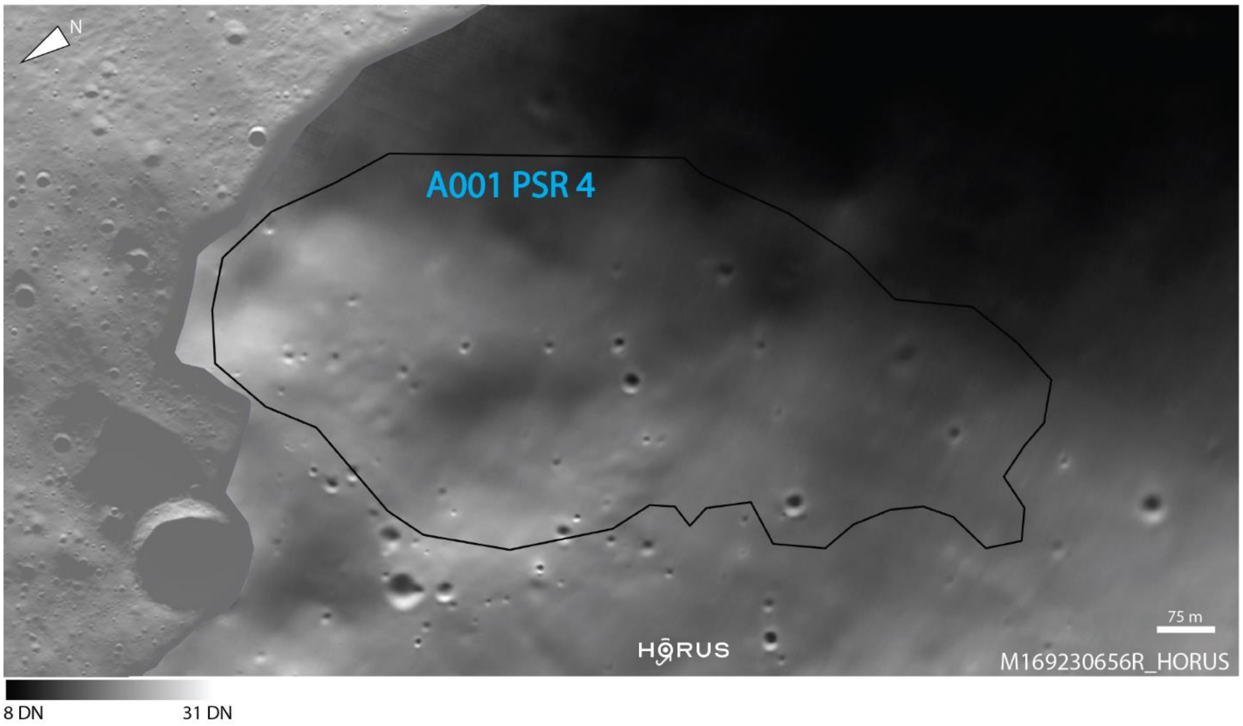

Figure S7 | AoI 001 PSR 4. View of the PSR (indicated by the black outline), NAC-observed TSR (indicated by the gray outline), and the sunlit surroundings. The most appropriate, currently available HORUS frame has been used to produce this figure. The sunlit part of the image is slightly transparent to help showcase the shadowed region. Raw NAC image credits to NASA/LROC/GSFC/ASU.

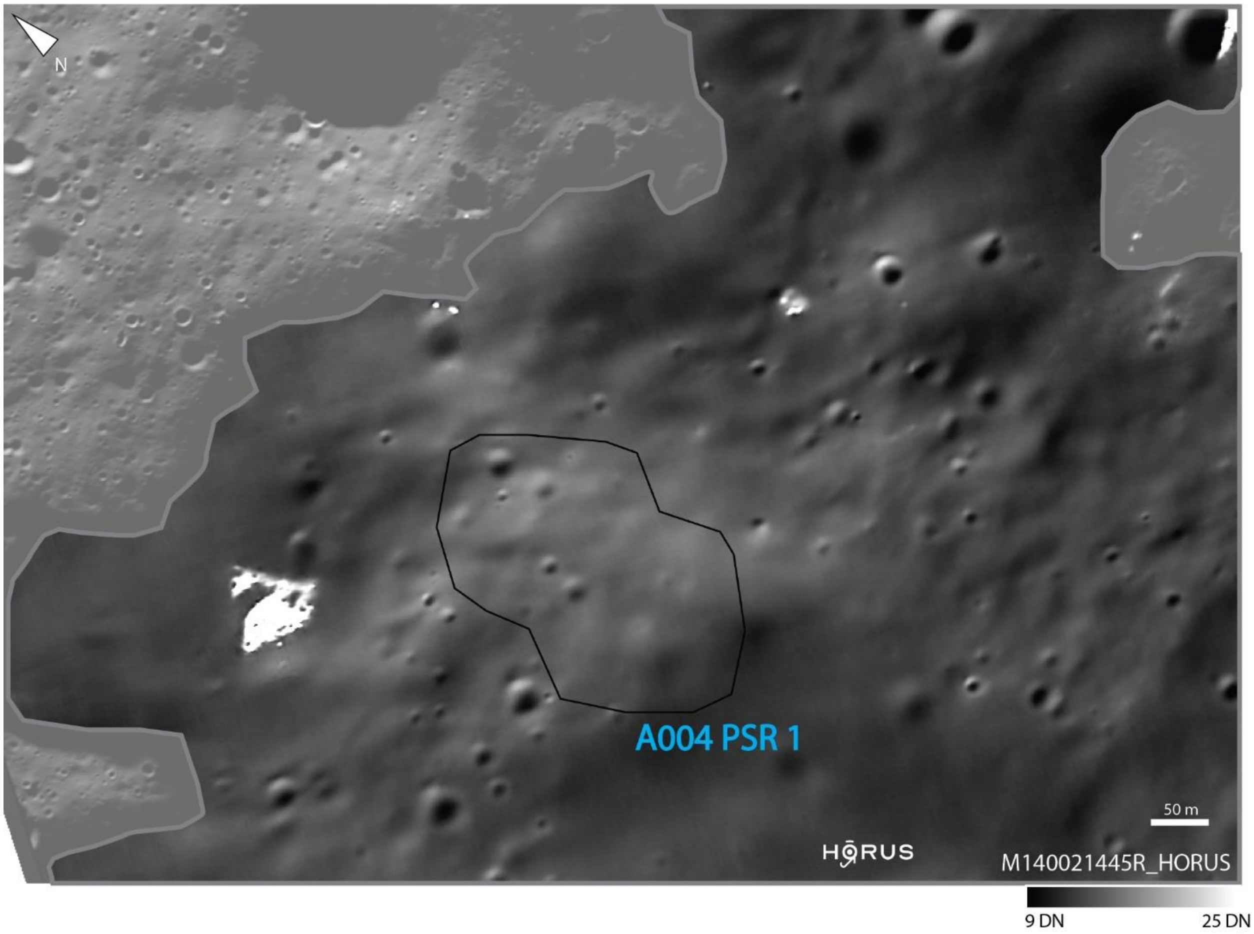

Figure S8 | AoI 004 PSR 1. View of the PSR (indicated by the black outline), NAC-observed TSR (indicated by the gray outline), and the sunlit surroundings. The most appropriate, currently available HORUS frame has been used to produce this figure. The sunlit part of the image is slightly transparent to help showcase the shadowed region. Raw NAC image credits to NASA/LROC/GSFC/ASU.

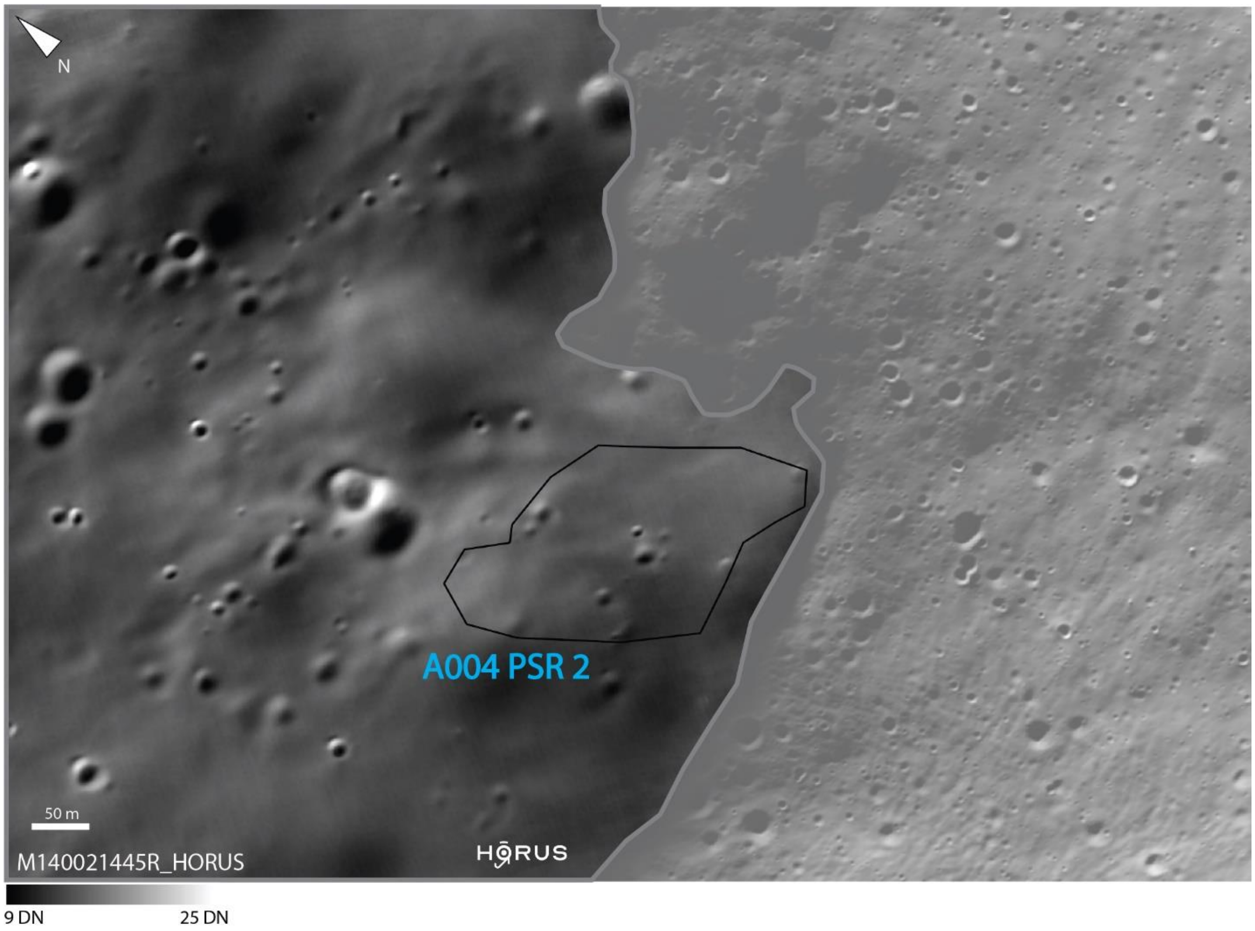

Figure S9 | AoI 004 PSR 2. View of the PSR (indicated by the black outline), NAC-observed TSR (indicated by the gray outline), and the sunlit surroundings. The most appropriate, currently available HORUS frame has been used to produce this figure. The sunlit part of the image is slightly transparent to help showcase the shadowed region. Raw NAC image credits to NASA/LROC/GSFC/ASU.

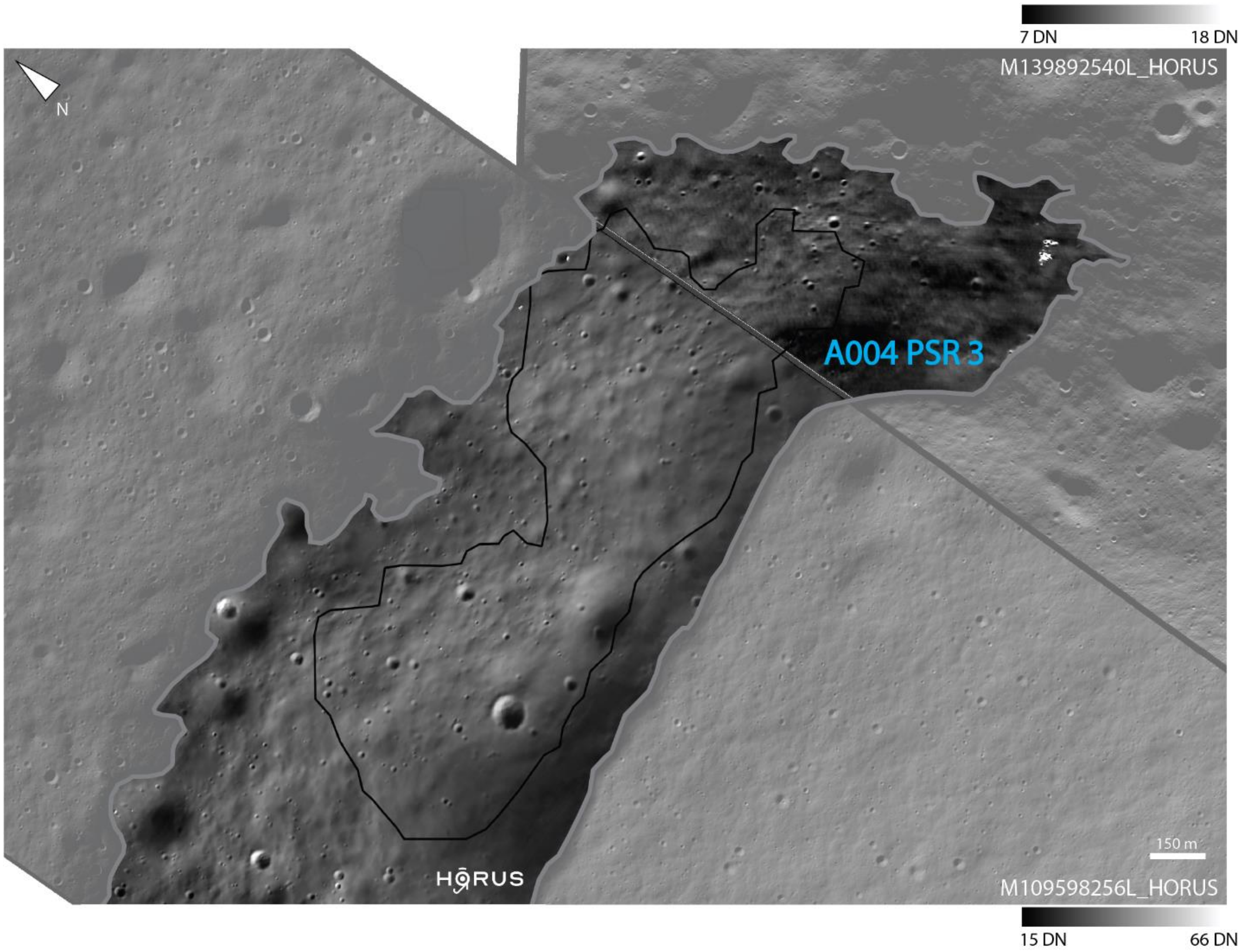

Figure S10 | AoI 004 PSR 3. View of the PSR (indicated by the black outline), NAC-observed TSR (indicated by the gray outline), and the sunlit surroundings. The most appropriate, currently available HORUS frame has been used to produce this figure. The sunlit part of the image is slightly transparent to help showcase the shadowed region. Raw NAC image credits to NASA/LROC/GSFC/ASU.

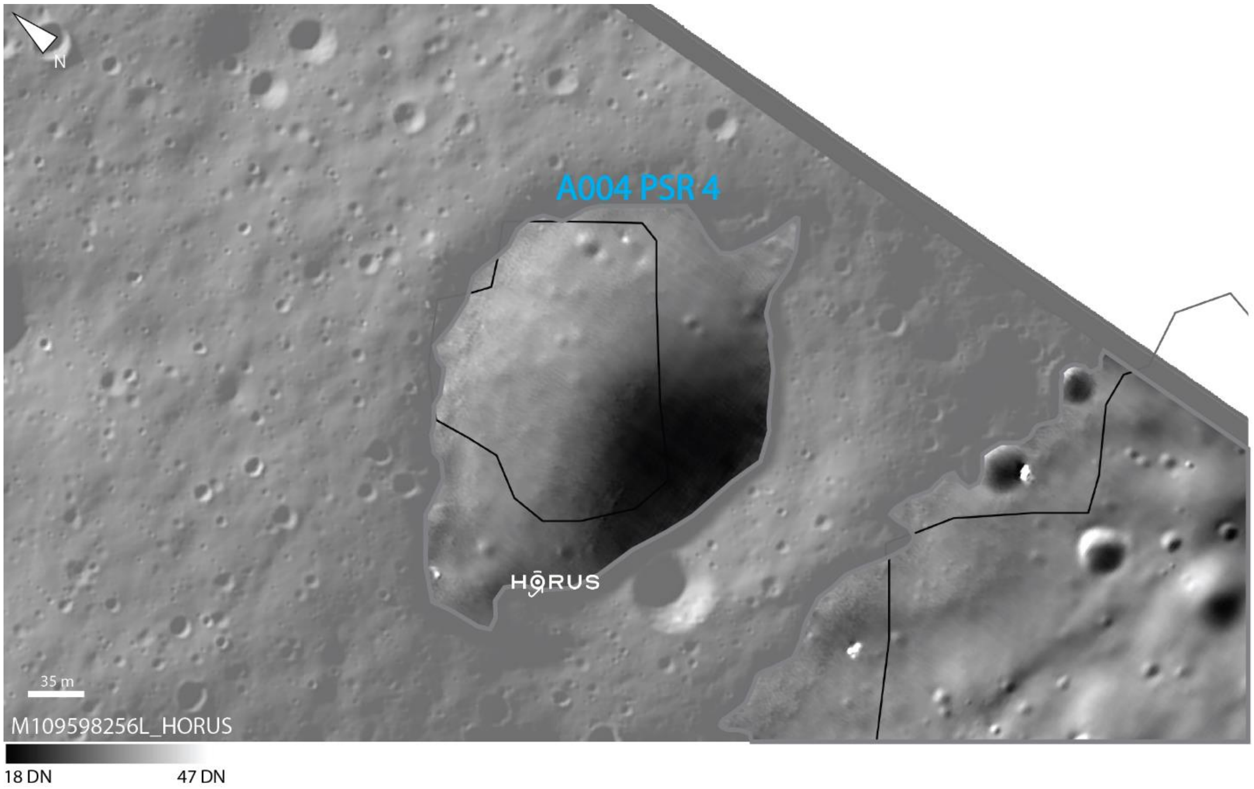

Figure S11 | AoI 004 PSR 4. View of the PSR (indicated by the black outline), NAC-observed TSR (indicated by the gray outline), and the sunlit surroundings. The most appropriate, currently available HORUS frame has been used to produce this figure. The sunlit part of the image is slightly transparent to help showcase the shadowed region. Raw NAC image credits to NASA/LROC/GSFC/ASU.

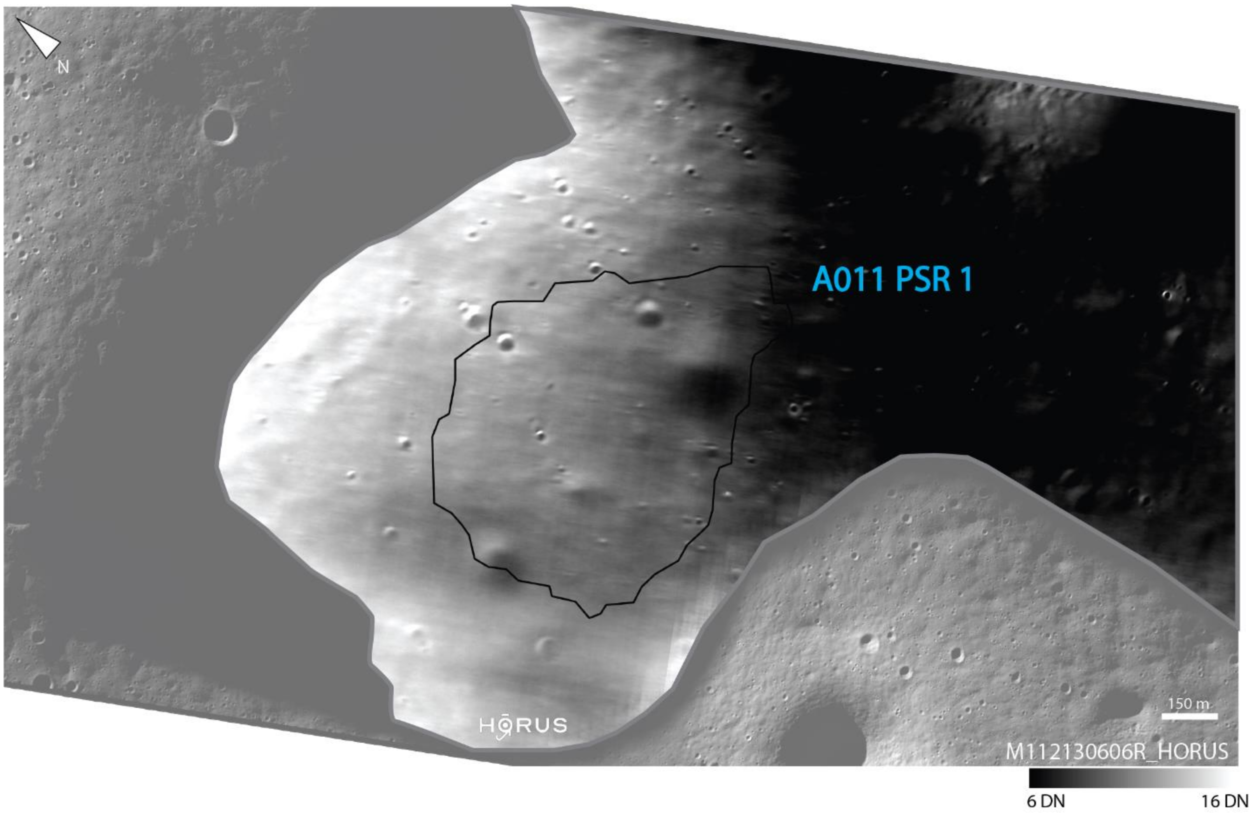

Figure S12 | AoI 011 PSR 1. View of the PSR (indicated by the black outline), NAC-observed TSR (indicated by the gray outline), and the sunlit surroundings. The most appropriate, currently available HORUS frame has been used to produce this figure. The sunlit part of the image is slightly transparent to help showcase the shadowed region. Raw NAC image credits to NASA/LROC/GSFC/ASU.

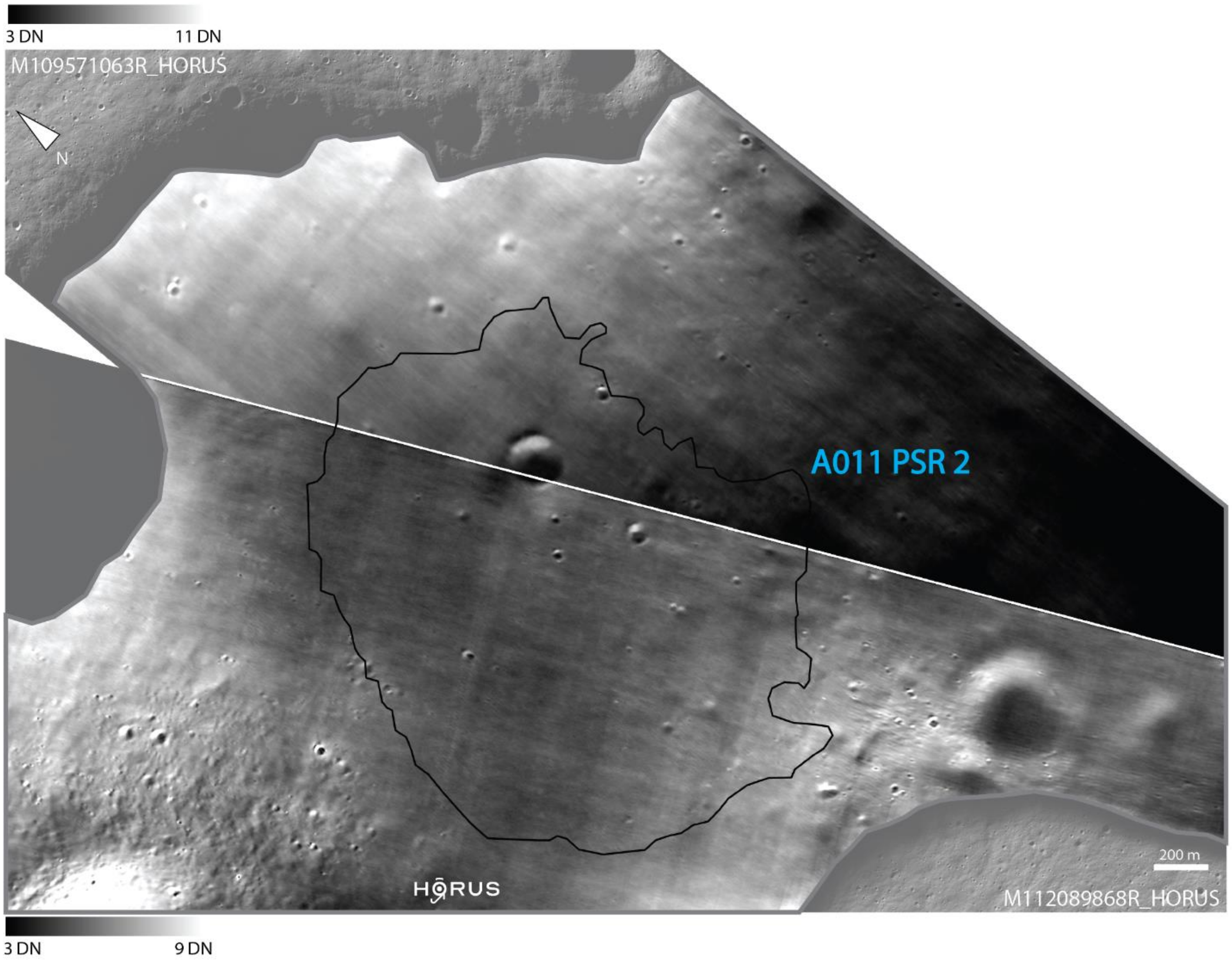

Figure S13 | AoI 011 PSR 2. View of the PSR (indicated by the black outline), NAC-observed TSR (indicated by the gray outline), and the sunlit surroundings. The most appropriate, currently available HORUS frame has been used to produce this figure. The sunlit part of the image is slightly transparent to help showcase the shadowed region. Raw NAC image credits to NASA/LROC/GSFC/ASU.

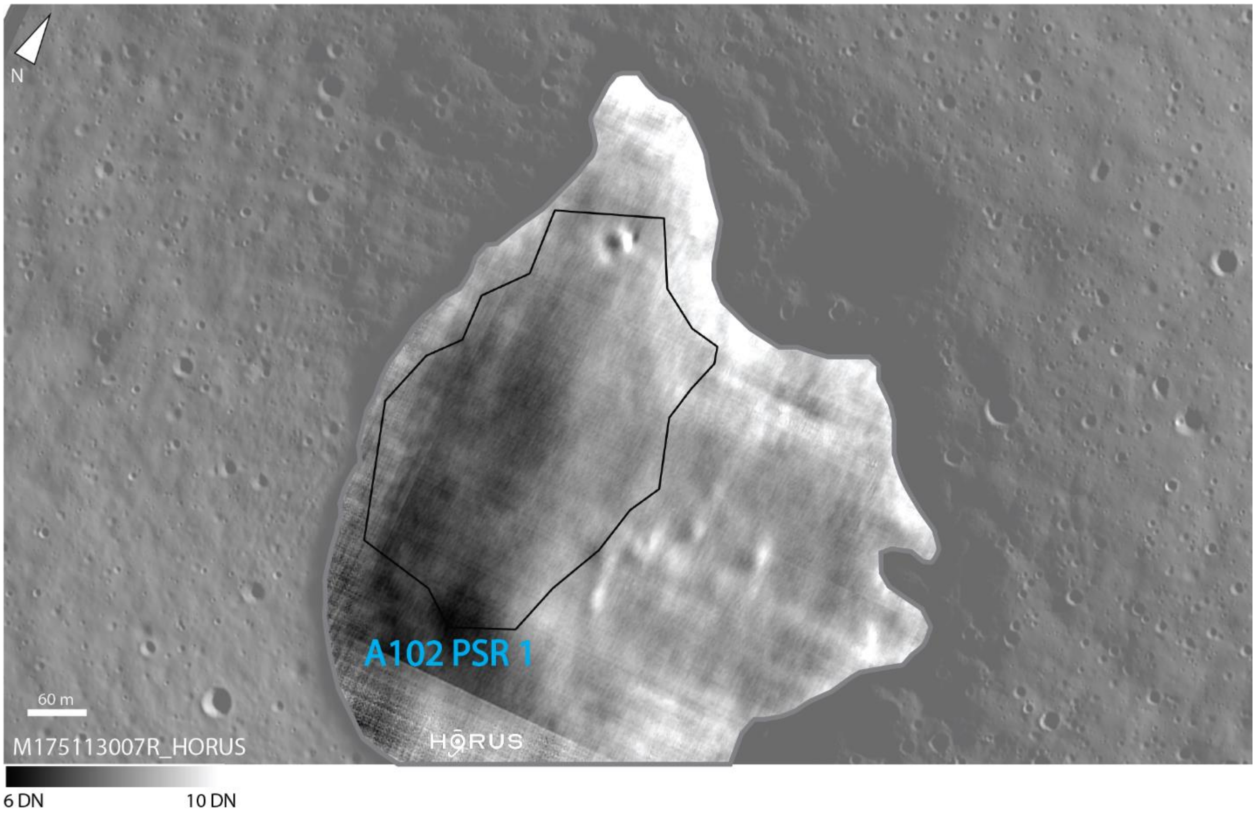

Figure S14 | AoI 102 PSR 1. View of the PSR (indicated by the black outline), NAC-observed TSR (indicated by the gray outline), and the sunlit surroundings. The most appropriate, currently available HORUS frame has been used to produce this figure. The sunlit part of the image is slightly transparent to help showcase the shadowed region. Raw NAC image credits to NASA/LROC/GSFC/ASU.

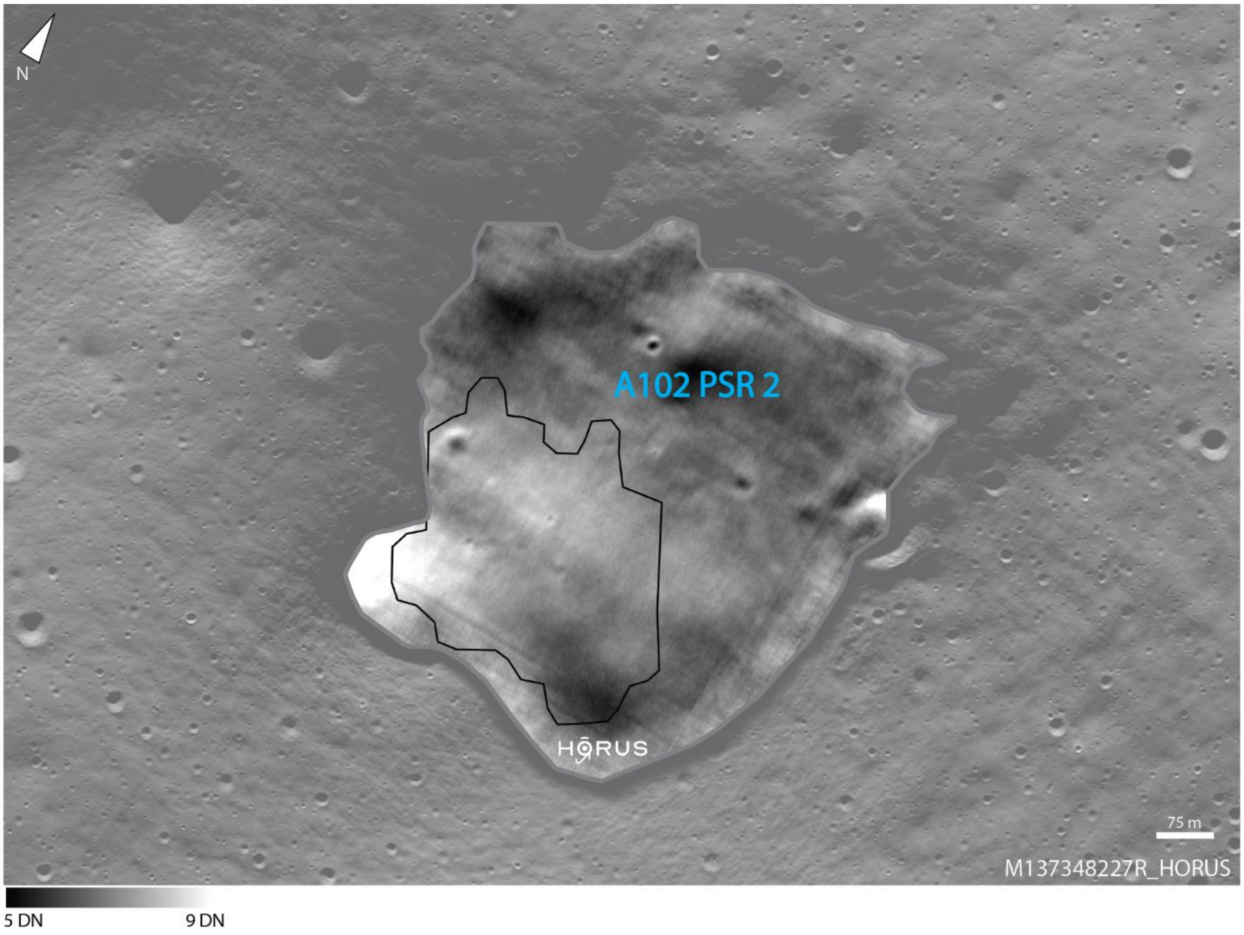

Figure S15 | AoI 102 PSR 2. View of the PSR (indicated by the black outline), NAC-observed TSR (indicated by the gray outline), and the sunlit surroundings. The most appropriate, currently available HORUS frame has been used to produce this figure. The sunlit part of the image is slightly transparent to help showcase the shadowed region. Raw NAC image credits to NASA/LROC/GSFC/ASU.

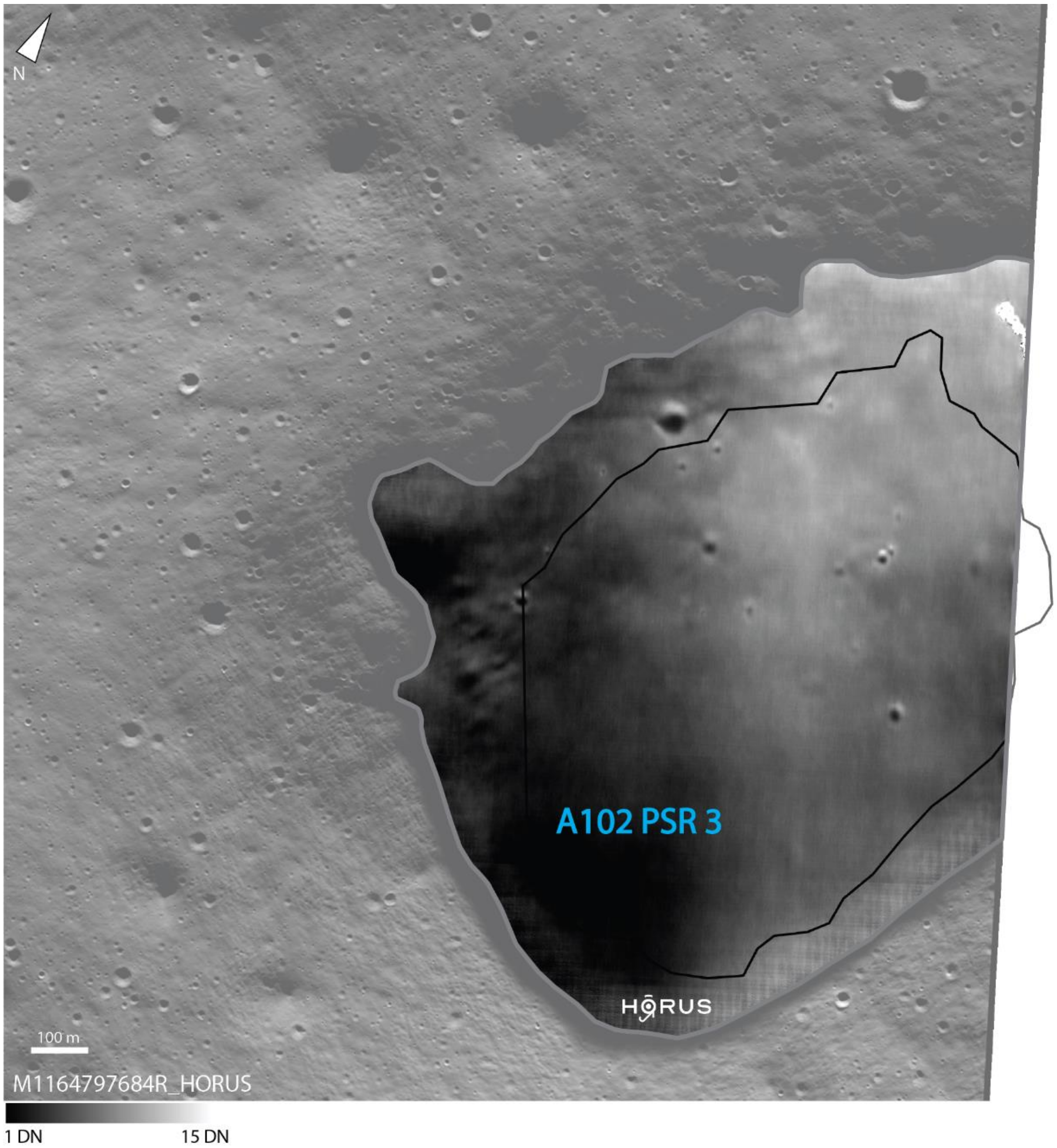

Figure S16 | AoI 102 PSR 3. View of the PSR (indicated by the black outline), NAC-observed TSR (indicated by the gray outline), and the sunlit surroundings. The most appropriate, currently available HORUS frame has been used to produce this figure. The sunlit part of the image is slightly transparent to help showcase the shadowed region. Raw NAC image credits to NASA/LROC/GSFC/ASU.

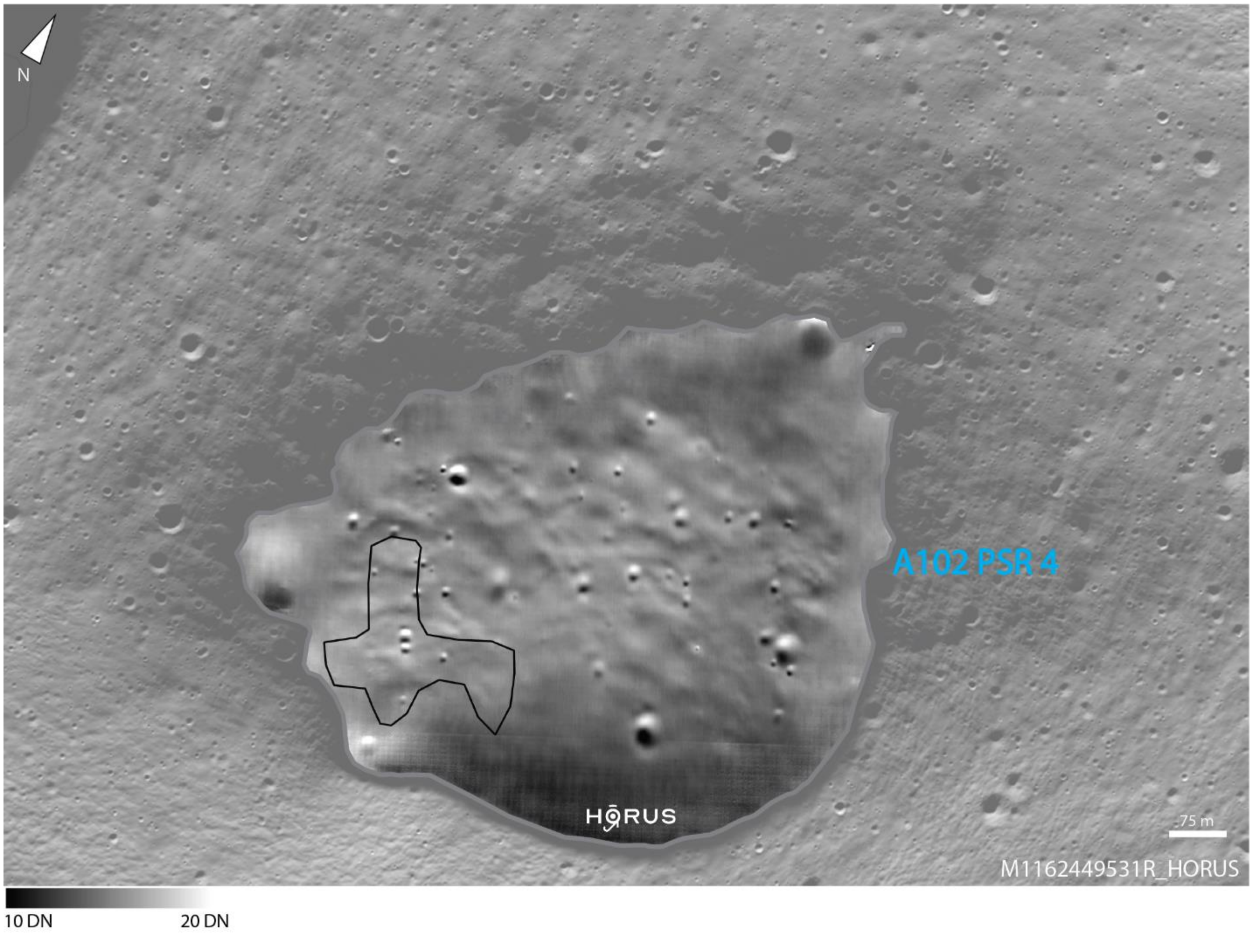

Figure S17 | AoI 102 PSR 4. View of the PSR (indicated by the black outline), NAC-observed TSR (indicated by the gray outline), and the sunlit surroundings. The most appropriate, currently available HORUS frame has been used to produce this figure. The sunlit part of the image is slightly transparent to help showcase the shadowed region. Raw NAC image credits to NASA/LROC/GSFC/ASU.

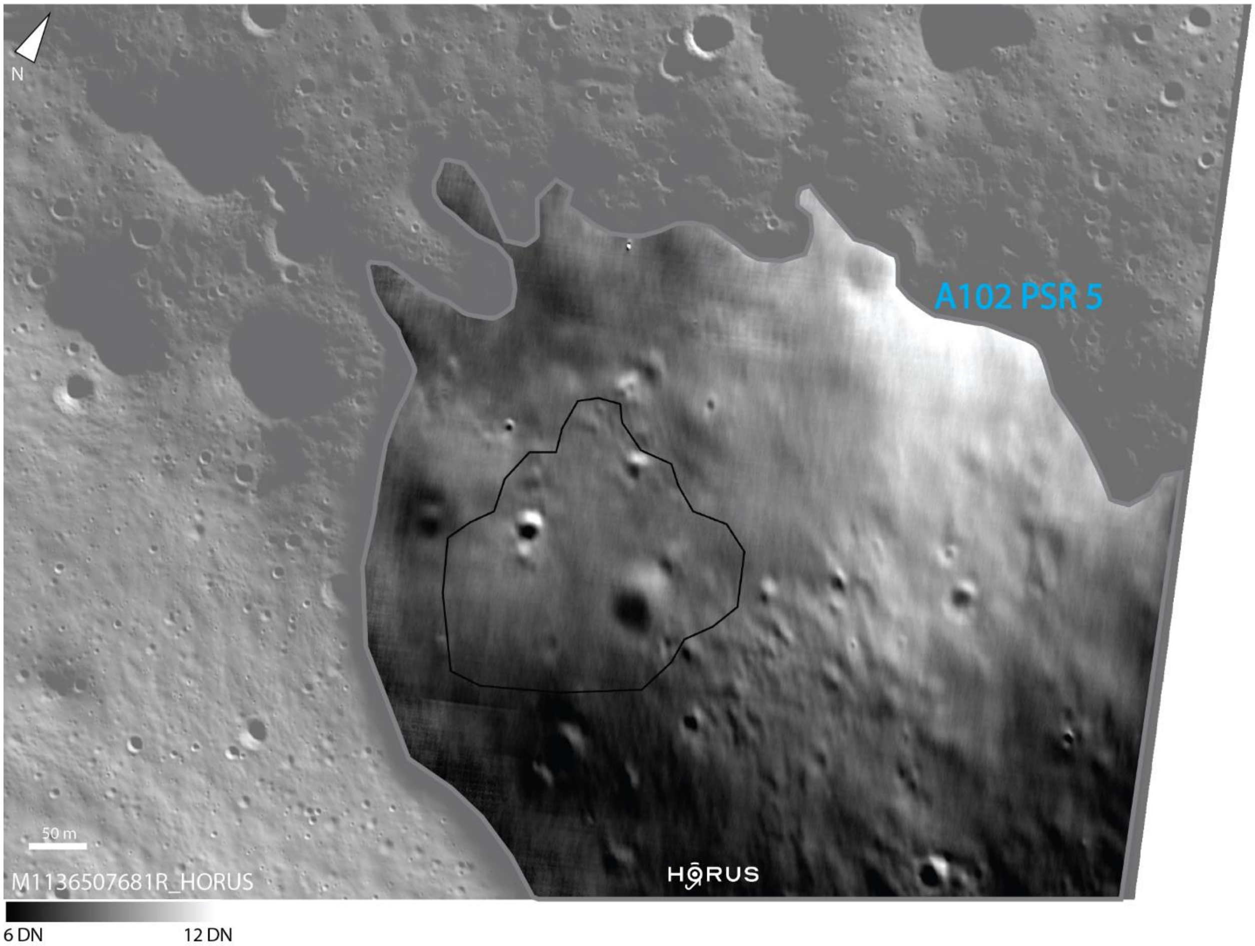

Figure S18 | AoI 102 PSR 5. View of the PSR (indicated by the black outline), NAC-observed TSR (indicated by the gray outline), and the sunlit surroundings. The most appropriate, currently available HORUS frame has been used to produce this figure. The sunlit part of the image is slightly transparent to help showcase the shadowed region. Raw NAC image credits to NASA/LROC/GSFC/ASU.

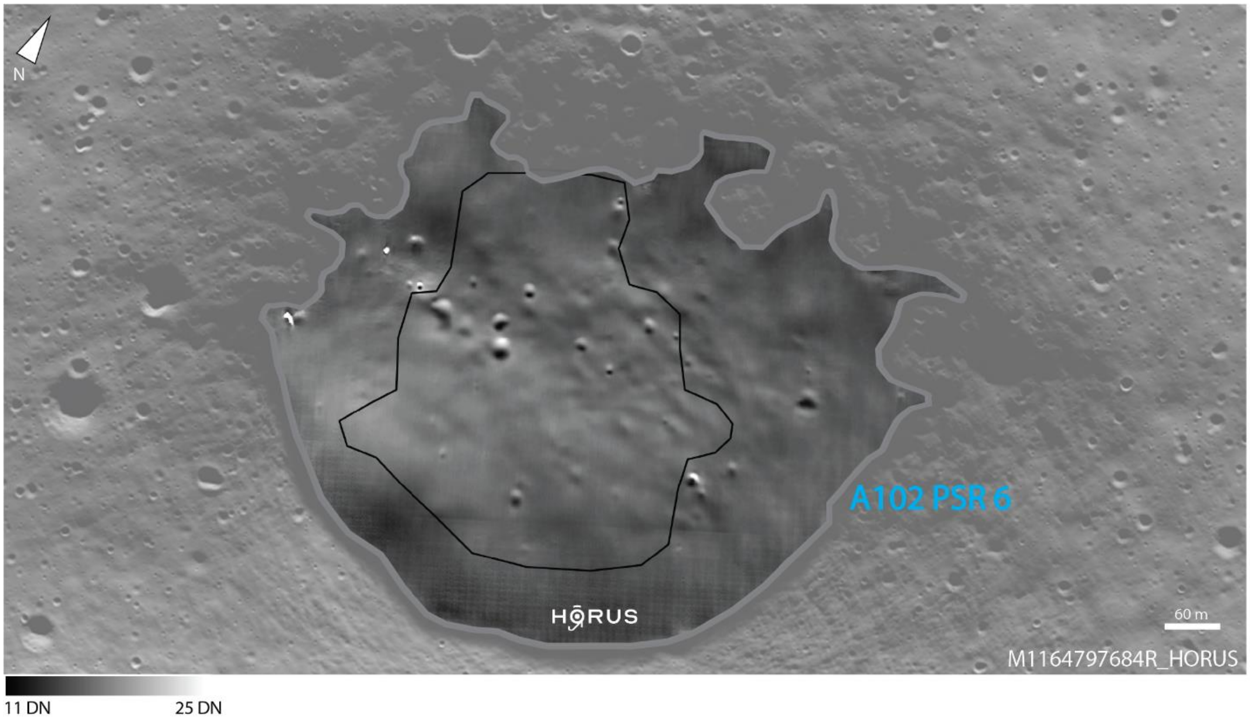

Figure S19 | AoI 102 PSR 6. View of the PSR (indicated by the black outline), NAC-observed TSR (indicated by the gray outline), and the sunlit surroundings. The most appropriate, currently available HORUS frame has been used to produce this figure. Please note that for this figure the boundary of the PSR has been derived from a 60 m PSR product (Mazarico et al., 2011), in contrast to Figure 5, for visualization purposes. The sunlit part of the image is slightly transparent to help showcase the shadowed region. Raw NAC image credits to NASA/LROC/GSFC/ASU.

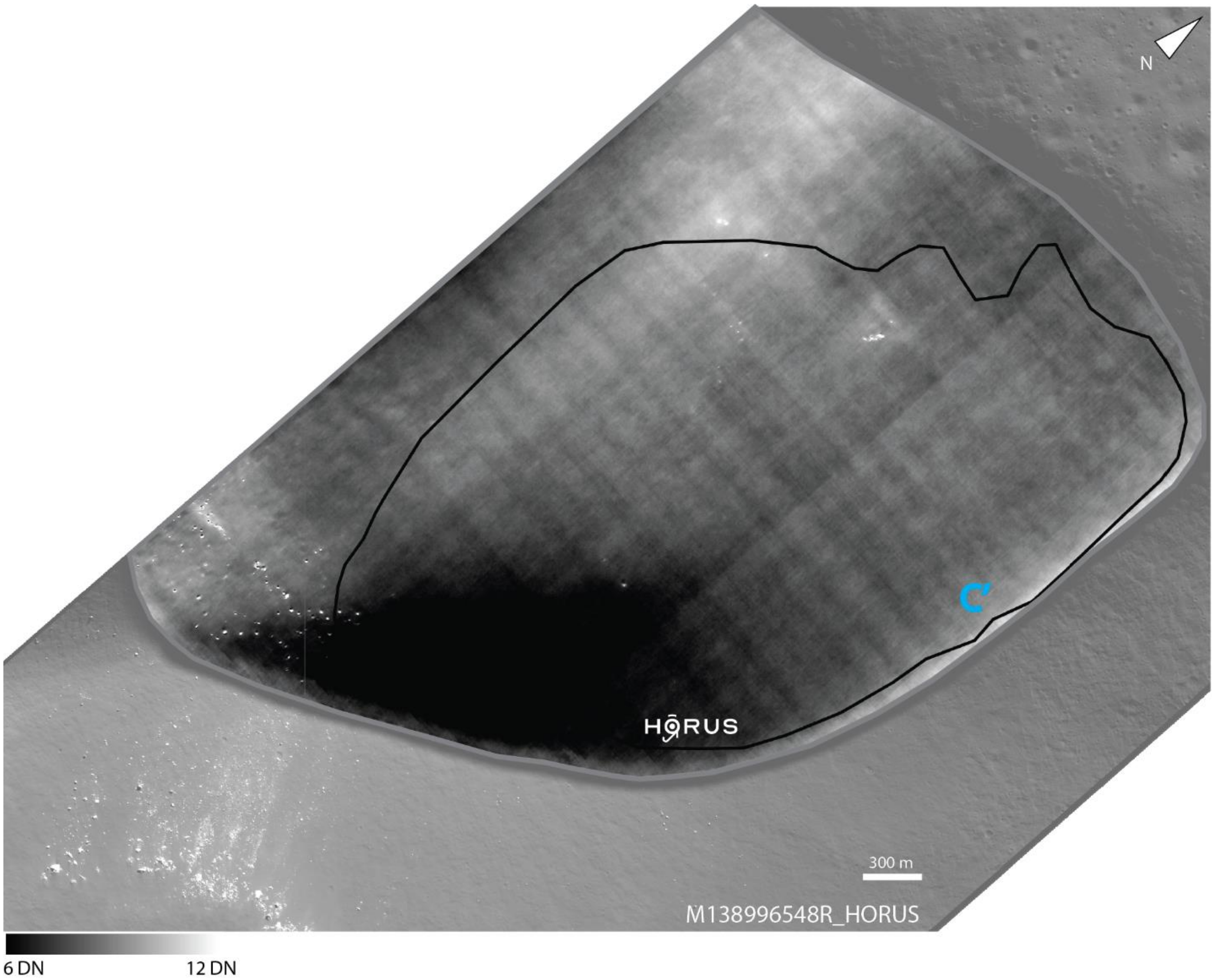

Figure S20 | AoI C' PSR. View of the PSR (indicated by the black outline), NAC-observed TSR (indicated by the gray outline), and the sunlit surroundings. The most appropriate, currently available HORUS frame has been used to produce this figure. Relevant geomorphic features are outline in Figure S21. The sunlit part of the image is slightly transparent to help showcase the shadowed region. Raw NAC image credits to NASA/LROC/GSFC/ASU.

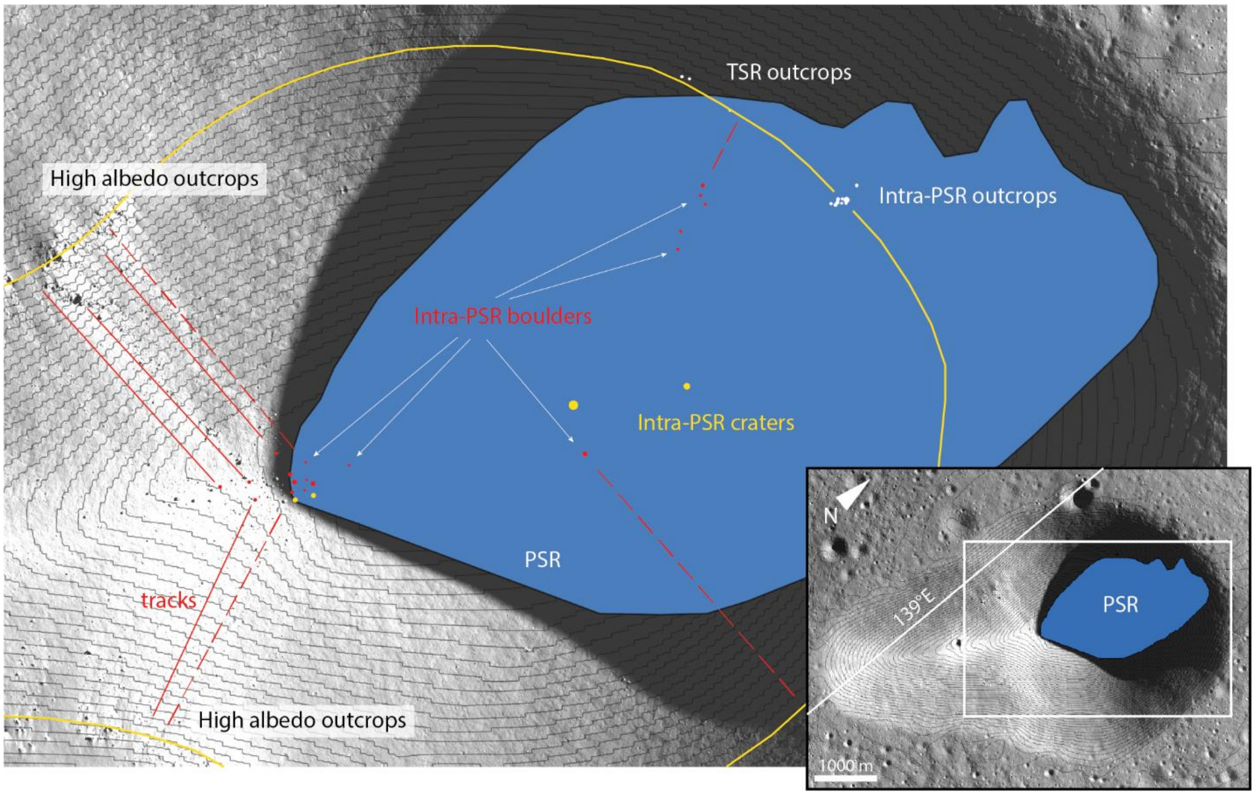

Figure S21 | Overview of AoI PSR C', Schrödinger Basin vent. Overview of the HORUS-mapped geomorphic features in the Schrödinger Basin pyroclastic vent; overview map outlines the spatial extent of the detailed map. The map shows regular (TSR) and intra-PSR outcrops (white dots), boulders (small red dots), and craters (small yellow dots), as well as rockfall trajectories (lines) that are observed (red continuous) or suggested (red dashed). The discovered intra-PSR outcrops are on the same topographic elevation (indicated with a yellow contour line) as the other high-albedo outcrops across the vent, potentially indicating they represent the same lithological unit. Please refer to Figure S20 for a view into the C' PSR (HORUS image). The vent PSR features an anomalously low density of craters; if not caused by the scarce secondary illumination, the apparent lack of craters in this PSR could mean that it is much younger than the sunlit portion of the vent as well as the surroundings, meaning there is an unknown process that has been re-working this particular PSR until today. However, we note that an observational bias is likely (see Figure S3). Raw NAC image credits to NASA/LROC/GSFC/ASU.

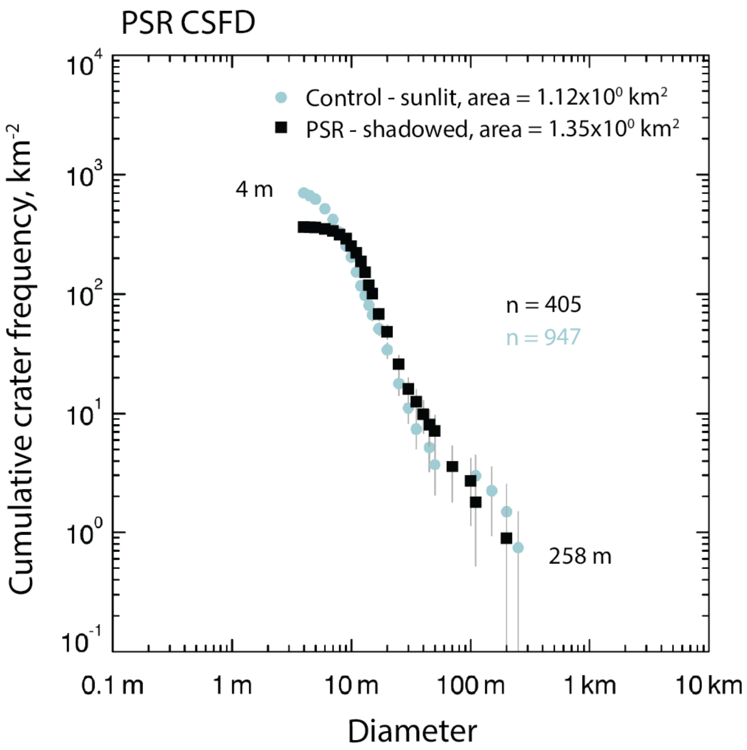

Figure S22 | Crater size frequency distributions (CSFD) for PSR A004 PSR 3 and its sunlit control site. Comparison of two cumulative CSFD curves (log-log plot), one derived in a sunlit control site (blue curve, M140007876RC; control site is ~300 m away from the PSR) and one derived in the PSR (black curve, M109598256L_HORUS). The curves suggest that the PSR features fewer craters with diameters <~8.5 m than the sunlit control region, which might point at a physical process that erases small craters or at anomalous regolith properties that result in increased crater diameters (e.g. high porosity). However, additional tests show that this effect is likely caused by an observational bias of HORUS (Figure 6). The error bars correspond to the statistical error of the cumulative numbers ($\sqrt{N}$).

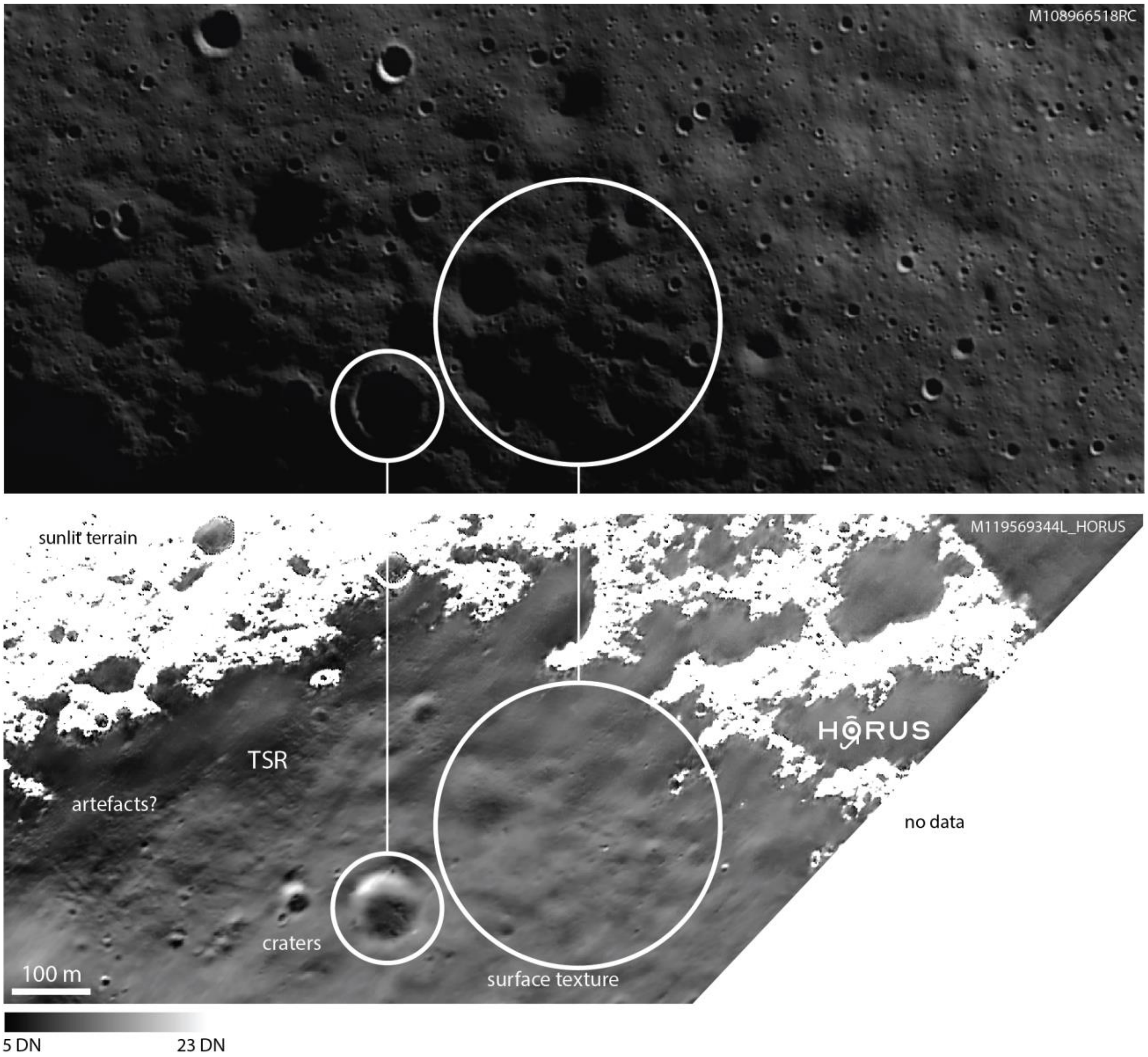

Figure S23 |TSR comparison of surface texture in sunlit and shadowed conditions. Qualitative analysis of the visual appearance of surface texture and lobate-type features in sunlit and shadowed conditions (HORUS) in a TSR close to A001 PSR 10 (LAT 89.59°S, LON 118.73°W). Overall – HORUS images appropriately display the surface texture and relevant features as observed in sunlit conditions. Proper multi-frame on-site image validation (Figures 3 and 4) allows to decrease the potential uncertainty related to varying illumination conditions and image artefacts. Raw NAC image credits to NASA/LROC/GSFC/ASU.

Table S1 | Summary of AoI PSR characteristics. Numeric characterization of the geomorphology and temperature conditions of all studied PSRs; A = Artemis, C = Constellation (please refer to Figure 2 for geographic details).

AoI PSR ID	Annual max T (K)	Annual mean T (K)	Area (km^2)	No. boulders	No. craters	% of PSR cratered	Crater per PSR km^2
A001_1	151	86	4.83	0	2	0.64	0.41
A001_2	154	79	1.55	0	5	1.59	3.22
A001_3	96	64	18.10	0	10	6.92	0.55
A001_10	160	95	3.46	0	34	2.23	9.82
A004_1	186	91	0.23	3	16	12.23	68.28
A004_2	183	91	0.18	0	11	6.56	60.06
A004_3	153	79	11.18	3	405	12.27	36.24
A004_4	202	94	0.34	0	2	1.65	5.81
A011_1	110	72	8.43	0	13	6.50	1.54
A011_2	121	69	33.76	0	15	1.53	0.44
A102_1	178	91	0.90	0	2	2.10	2.23
A102_2	184	92	1.44	0	2	1.20	1.39
A102_3	159	77	5.70	0	13	1.35	2.28
A102_4	198	106	0.25	0	9	5.29	36.18
A102_5	232	104	0.62	0	6	10.60	9.68
A102_6	214	102	1.08	0	28	5.52	25.94
C’	168	94	53.82	14	4	0.03	0.07
